# Parto humanizado y enfoque diferencial de derecho: percepción de la puérpera adolescente. Montería, Colombia

**DOI:** 10.15446/rsap.V25n4.107259

**Published:** 2023-07-01

**Authors:** Ariadgna Gilma Castro-Morales, Eva María López De La Espriella

**Affiliations:** 1 AC: Enf. M. Sc. Salud Pública. Universidad de Córdoba. Montería, Colombia. ariadgnacastrom@correo.unicordoba.edu.co Universidad de Córdoba Salud Pública Universidad de Córdoba Montería Colombia ariadgnacastrom@correo.unicordoba.edu.co; 2 EL: Enf. M. Sc. Salud Pública. Universidad de Córdoba. Montería, Colombia. evalopezd@correo.unicordoba.edu.co Universidad de Córdoba Salud Pública Universidad de Córdoba Montería Colombia evalopezd@correo.unicordoba.edu.co

**Keywords:** Percepción, puérperas, parto humanizado, enfoque diferencial, aten ción *(fuente: DeCS, BIREME)*, Perception, puerperal women, humanized childbirth, differential approach, care *(source*, MeSH, NLM)

## Abstract

**Objetivo:**

Evaluar la percepción de la puérpera adolescente relacionada con la aten ción del parto humanizado, desde el enfoque diferencial de derecho.

**Metodología:**

Se realizó un estudio con enfoque cuantitativo, descriptivo, retrospectivo y transversal en el que se recolectaron los datos en una encuesta aplicada a 60 puér peras que tuvieron sus hijos durante el año 2022, en una institución de Salud de la ciudad de Montería, Colombia.

**Resultados:**

La edad más frecuente del parto son los 17 años, con un 50%; el 83% residía en Montería, el 98% pertenecían al estrato socioeconómico 1, el 95% tenía estudios secundarios incompletos, el 32% se identificó como mestiza y el 90% eran madres primerizas. De igual forma, el 77% manifestó haber vivido el momento del parto de manera tranquila, y el 85% señaló no haber recibido información relacionada con el parto humanizado.

**Conclusiones:**

Se evidenció que la institución de salud donde se aplicó el estudio no ofrecía la atención del parto humanizado, que es el anhelo de las gestantes, las cuales quieren vivir este importante momento en compañía de su pareja y su familia. Se encontraron deficiencias en el trato recibido por parte del personal de salud que atendió el parto.

## Abstract (en)

**Objective:**

To evaluate the perception of the adolescent puerpera related to humanized delivery care from the differential legal approach.

**Methodology:**

A study was carried out with a quantitative, descriptive, retrospective and cross-sectional approach where data was collected in a survey applied to 60 puerperal women, who had their children during the year 2022, in a Health institution in the City of Montería, Colombia.

**Results:**

The most frequent age of delivery is 17 years with 50%; 83% resided in this city of Montería, 98% belonged to socioeconomic stratum 1, 95% had incomplete secondary studies, 32% identified themselves as mestizo, and 90% were first-time mothers. Similarly, 77% stated that they had experienced the moment of childbirth calmly and 85% stated that they did not receive information related to humanized childbirth.

**Conclusions:**

It was evidenced that the health institution where the study was applied did not offer humanized delivery care, this situation being desired by pregnant women who want to live this important moment, in the company of their partner and family. Deficiencies were found in the treatment received by the health personnel who attended the delivery.

El parto es un momento único e importante en la vida de la mujer y su recién nacido, es un proceso fisiológico, durante el cual la madre le pone fin a la tan anhelada espera 1, en la cual la mujer perdió protagonismo 2, en el momento en que es ella quien tiene que adaptarse a los protocolos existentes en las diferentes instituciones de salud, y no los profesionales de la salud ponerse al servicio de esa atención individua lizada 3. También es importante resaltar que, durante la atención del parto, la mujer es vulnerable y puede ser víctima de violencia obstétrica, la cual es definida por Gabriela Almonte García como toda conducta, acción u omisión realizada por el personal de salud que afecta de manera directa o indirecta el cuerpo de la mujer y los procesos reproductivos de esta, lo cual se expresa como un trato deshumanizado 4. Para disminuir esta brecha de desigualdad, se abre espacio a la atención del parto con enfoque diferencial de derechos, donde el parto humanizado toma en cuenta las emociones, los senti mientos y los derechos, no solo de la mujer, sino de su familia, durante la atención del embarazo, la atención del parto y el puerperio, esto con el fin de que la mujer retome el protagonismo en el parto humanizado a tomar decisiones sobre dónde, cómo y con quién parir 5.

Los profesionales de la salud cumplen un papel funda mental en la atención del parto humanizado, toda vez que son los que brindan los cuidados y quienes tienen la posibilidad de garantizar un parto que incluya toda la esfera humana, donde se distingue el concepto de parto normal medicalizado con relación al parto natural sin ninguna práctica instrumentalizada 6. Por lo anterior, es importante que el profesional de la salud garantice que se vele por los derechos de la mujer durante el parto, que se le brinde un trato digno, libre de conductas abusivas y maltratantes que den pie a la violencia obstétrica, al igual que se respeten los planes que tiene la madre con relación al parto, se promueva una libre posición durante el trabajo de parto, se respete el derecho de la futura madre a perma necer acompañada y apoyada en todo momento durante la labor del parto, y que cuente con información clara y transparente sobre el transcurso de su labor, de manera que se permita una humanización del cuidado 7.

En relación con la puérpera adolescente, es importante mencionar que esta deja de ser objeto de cuidado de sus padres para convertirse en madre, y asume la responsa bilidad de los cuidados tanto propios como de su recién nacido. En la mayoría de los casos, dichos cuidados son asumidos por su familia, debido a la inexperiencia de las recientes madres, su escasa edad y las costumbres familiares que se han transmitido de generación en generación 8.

Es importante mencionar que muchas de estas puérperas adolescentes están enmarcadas dentro del enfoque diferencial de derechos, donde se les debe reconocer desde su individualidad, teniendo en cuenta sus derechos colec tivos constitucionales, tomando como criterio preferencial la protección al individuo desde la colectividad. Es aquí donde la atención en salud se convierte en diferencial, teniendo en cuenta las características del sujeto de atención, las familias y las comunidades 9.

Asimismo, la atención en salud ha tratado de recuperar su esencia: "la humanización del servicio", donde la sinergia que existe entre los conocimientos científicos y los valores rescata la sensibilización en la prestación de servicio, al centrarse en el paciente y basarse en una atención integral y consecuente con los derechos colec tivos y constitucionales del individuo 10.

En Colombia, con la Resolución 2626 de 2019, se imple mento el Modelo de Atención Integral en Salud (MAITE), el cual pretende integrar elementos que proporcionen el desarrollo de herramientas en el marco de sus ocho líneas de acción, siendo una de estas líneas el enfoque diferencial, en el que se reconoce que hay poblaciones con características particulares, las cuales se relacionan con su edad, el género, la etnia y la condición de discapacidad o de víctimas de la violencia, entre otras circunstancias que las ubican en situación de desventaja y vulnerabilidad al momento de recibir la atención en salud. Por tanto, es necesario adaptar la atención en salud a las características particulares y colectivas, de forma que se minimicen las desigualdades y las inequidades existentes del sistema de salud actual 11.

Con base en el cumplimiento de la Resolución 2626, con énfasis en la línea de enfoque diferencial, las gestantes quieren ser atendidas en instituciones de salud que velen por garantizar una atención humanizada, en el contexto de un parto humanizado. Por ello, se busca revisar cómo se atiende a las gestantes en una institución de salud de la ciudad de Montería (Colombia), teniendo en cuenta que en esta modalidad de parto apenas se está implemen tando en algunas instituciones de salud.

## METODOLOGÍA

Se realizó un estudio con enfoque cuantitativo, descriptivo, retrospectivo y transversal, en el cual se midieron variables demográficas y se analizó el nivel de percepción del parto humanizado. Los datos se obtuvieron de una encuesta aplicada con previo consentimiento de los padres o cuida dores de las 60 puérperas adolescentes a las que se les brindó la atención del parto durante el año 2022 en una institución de salud de tercer nivel en la ciudad de Montería (Colombia), a quienes se les explicó el objetivo de la inves tigación y la garantía de anonimato y confidencialidad de la información. Las mujeres fueron seleccionadas mediante muestreo no probabilístico por conveniencia en la medida en que eran atendidas, a partir de los siguientes criterios: a) puérperas adolescentes entre 12 y 17 años; b) con atención del parto vaginal institucional; y c) que desearan participar en el estudio. Como criterios de exclusión se tuvieron los siguientes: a) gestantes adolescentes; b) con atención del parto por cesárea o instrumentado; y c) que no desearan participar en el estudio.

Para el estudio se entrevistó a 60 puérperas adoles centes que vivieron la experiencia del parto institucional. Las participantes diligenciaron una encuesta semiestructurada que contenía 23 ítems, en la que se indagó en torno a tópicos como el lugar de residencia, el estrato socioeco nómico, la edad, el grupo étnico con el cual se identifican, el nivel educativo y el número de partos. De igual manera, se indagó sobre la experiencia que tuvieron al momento del parto, la percepción de la atención que recibieron en la institución de salud, el acceso a un acompañante durante el momento del parto y si compartirían su experiencia con otras mujeres. Los resultados fueron recolectados, digitalizados y organizados en una base de datos con la herramienta de Microsoft Excel y posteriormente fueron analizados con el *software* estadístico IBM SPSS Statistics Base 22.0 para Windows.

La participación de las puérperas adolescentes en el estudio fue voluntaria, con previo consentimiento escrito de sus padres o cuidadores; la información recolectada mediante el instrumento de evaluación se manejó de forma confidencial y anónima, con previo consentimiento informado. Para el diseño y la ejecución de la investigación, se tuvieron en cuenta los lineamientos éticos y deontológicos para estudios con seres humanos estipulados en la Declaración de Helsinki 12 y las normas científicas, técnicas y administrativas para la investigación en salud, reglamentadas según la Resolución 8430 de 1993 del Ministerio de Salud y Protección Social colombiano 13.

## RESULTADOS

En el estudio participaron 60 puérperas adolescentes, quienes vivieron la experiencia del parto institucional en una entidad de Montería (Colombia), cuya edad fue de 14 a 17 años. Del análisis de los datos surgen los siguientes hallazgos: el 50% de la población estuvo constituida por puérperas con 17 años, residentes en la ciudad de Montería en un 83%, entre las que destaca un 67% de residentes en la zona urbana de la capital cordobesa; el 98% de estas pertenece al estrato socioeconómico 1. Por otra parte, se evidencia que el 95% de las participantes habían logrado cursar estudios secundarios incompletos, mientras que el 32% se identificó como perteneciente a la raza mestiza y el 90% eran primigestantes (Tabla 1).

**Tabla 1:** Características sociodemográficas de las puérperas adolescentes con atención de parto en una institución de salud del municipio de Montería, Colombia - año 2022 (n=60)

Variables	N.°	%
Edad		
14	5	8
15	10	17
16	15	25
17	30	50
Municipio de residencia		
Cereté	3	5
Ciénaga de Oro	1	2
Cotorra	1	2
Medellín	1	2
Montería	50	83
Puerto Escondido	3	5
San Pelayo	1	2
Zona de residencia		
Rural	20	33
Urbano	40	67
Estrato socioeconómico		
1	59	98
2	1	2
Nivel educativo		
Primaria	3	5
Secundaria	57	95
Grupo étnico		
Afro	2	3
Indígena	3	5
Mestiza	19	32
Ninguno	36	60
Número de partos		
1	54	90
2	6	10

Con respecto a la percepción de las puérperas adoles centes sobre la atención de parto recibido, es importante mencionar que al 85% de la población no se le permitió escoger al profesional ni a la institución donde sería atendido, como tampoco se le brindó información sobre el parto humanizado y el tipo de atención que recibiría (55%). Por otra parte, el 77% manifestó haber vivido el parto institucional de manera tranquila, el 45% conocía sus derechos en relación con la atención del parto, mientras que un 15% manifestó haber recibido información relacionada con el parto humanizado en la institución de salud.

En cuanto a la disponibilidad de elementos de apoyo (90%), medidas de confort (97%) y disponibilidad de un acompañante durante el trabajo de parto (95%), la gran mayoría manifestó que no contó con estos. Así mismo, el 77% aseguró que se le protegió su intimidad, y se le permitió tener libertad de movimiento durante el parto al 55%. El 73% exteriorizó que inmediatamente después del nacimiento les acercaron a sus hijos para el contacto piel a piel, y el 50% pudo iniciar la lactancia materna dentro de la primera hora del nacimiento. Lo anterior muestra que en la ciudad de Montería aún se aplica el modelo convencional medicalizado para la atención del parto, lo cual no permite a las parturientas que este momento sea vivenciado con los principios que plantea la Organización Mundial de la Salud (OMS), donde el parto humanizado arraiga importancia a nivel emocional, cultural y social para la mujer y su familia. Es importante mencionar además que el tipo de atención ofrecida en la ciudad no se asemeja a la planteada por el parto humanizado, donde el foco es la atención acogedora y segura para la paciente, con respeto de sus derechos, elecciones e individualidad (Tabla 2).

**Tabla 2:** Percepción de las puérperas adolescentes sobre la atención de parto recibido en una institución de salud del municipio de Montería (Colombia) - año 2022 (n=60)

Variables	Sí	%	No	%
Se le permitió elegir el profesional y la institución de salud donde tendría el parto	9	15	51	85
Se le explicó el tipo de parto o atención que iba a recibir	27	45	33	55
Al momento del parto conocía sus derechos con relación a la atención que debía recibir	27	45	33	55
Se le ofreció información relacionada con el parto humanizado	9	15	51	85
Vivió el momento del parto de manera tranquila	46	77	14	23
Se le permitió libertad de movimiento durante el trabajo de parto	33	55	27	45
Contó con elementos de apoyo durante el trabajo de parto	6	10	54	90
Contó con medidas de confort durante el trabajo de parto	2	3	58	97
Se le permitió contar con un acompañante en el momento del parto	3	5	57	95
Se le garantizó privacidad e intimidad en el trabajo de parto	46	77	14	23
Se le colocó al bebé sobre el pecho para contacto piel con piel	44	73	16	27
Inició la lactancia materna dentro de la primera hora del nacimiento	30	50	30	50

Con relación a los aspectos que las participantes cambiarían de la atención recibida durante el parto, se pudo apreciar que el 26,7% manifestó que le gustaría contar con la compañía de un familiar o persona signi ficativa durante la vivencia de esta etapa; un porcentaje poco significativo, teniendo en cuenta la desinformación con relación al parto humanizado y la importancia que tiene. También es apreciable que un 38,3% manifestó que no cambiaría nada de la atención recibida, lo cual puede estar relacionado con la idealización que se tiene del tipo de atención que se recibe en los centros asistenciales de la ciudad, donde se considera que lo realmente importante es ser atendido por profesionales, sin importar si esta es de calidad y humanizada.

De igual modo, es importante mencionar que las puérperas manifestaron que les gustaría mejorar la atención recibida por parte del personal médico (15%) y de enfermería (3,3%), lo cual puede estar relacionado con la violencia obstétrica que se ha evidenciado a lo largo de la historia en nuestro país, y más cuando se trata de adolescentes. Según estudios realizados por Zacher-Dixon 14, en Venezuela usuarias de los servicios obstétricos manifiestan algún tipo de deshumanización por parte del personal de salud, donde el trato deshumanizante se identificó por comentarios descalificadores, irónicos, sobrenombres, chistes o críticas por llorar o gritar, lo cual no es lejano a lo que manifestaron las usuarias encuestadas en el presente estudio (Tabla 3).

**Tabla 3:** Aspectos que cambiaría de la atención recibida durante el parto en la institución de salud del municipio de Montería (Colombia) - año 2022 (n=60)

Variables	N.°	%
No cambiaría nada, estuve satisfecha con la atención	23	38,3
Contar con un acompañante durante el parto	16	26,7
Mejorar la atención recibida por el personal médico	9	15,0
Parto sin dolor o cesárea	4	6,7
Experiencia dolorosa, perdí mi bebe	2	3,3
Mejorar la atención recibida por el personal de enfermería	2	3,3
Poder caminar, usar pelotas y ducha caliente	1	1,7
Que el parto sea asistido por personal femenino y que garanticen privacidad	1	1,7
Que no me picaran para el parto y eso me dejo con dolor	1	1,7
No presentar desgarro y dolor	1	1,7
Total	60	100

Fuente: Base de datos del estudio.

## DISCUSIÓN

Para conocer la percepción de la puérpera adolescente en relación con la atención del parto humanizado en una institución de salud de tercer nivel en la ciudad de Montería (Colombia), durante el año 2022, se realizó un análisis cuantitativo y descriptivo, para lo cual se aplicó una encuesta transversal a 60 puérperas que aceptaron participar libremente y cumplieron con los criterios de inclusión. La encuesta aplicada estuvo conformada por preguntas que les permitió a las investigadoras identificar aspectos sociodemográficos sobre las participantes, así como los conocimientos que tenían en relación con sus derechos, la atención del parto humanizado y recomenda ciones que darían a la institución de salud para posteriores atenciones en relación con el parto humanizado.

En ese sentido, el estudio se enfocó en la puérpera adolescente, porque es de interés de las autoras conocer cuál fue la experiencia que estas tuvieron en relación con la atención del parto humanizado, y si esta fue prestada desde el enfoque diferencial de derechos en la insti tución de salud. En cuanto a literaturas de referencia para comparar los resultados, existe muy poca al respecto, pero algunas investigaciones permitieron orientar el análisis.

En relación con las características sociodemográficas, la edad con mayor predomino en el estudio fueron puérperas con 17 años cumplidos, que representaron un 50%, seguidas de aquellos con 16 años con el 25%, lo que evidencia que en Colombia el inicio de las relaciones sexuales en jóvenes es cada vez más temprano. Ello concuerda con los resultados obtenidos en el estudio titulado "Transitando la adolescente por la adolescencia", de Luz Angela Argote 8. El 83% de estas puérperas residían en el municipio de Montería, capital del departa mento de Córdoba, donde se concentra la mayor parte de las instituciones prestadoras de los servicios de salud en esa zona del país. En cuanto al nivel educativo, la población objeto de estudio contaba con nivel de básica secundaria incompleta, lo que está estrechamente relacionado con el curso de vida en el que están enmarcados los adoles centes en Colombia. Estos resultados se relacionan con el estudio mencionado, en el que la población estuvo consti tuida por 16 adolescentes puérperas, quienes coinciden con la población objeto de este estudio en las altas tasas de fecundidad en este curso de vida.

La mayor parte de este grupo poblacional no se identificó con ningún grupo étnico, lo que representa el 60%, seguido del 32% que se identificó con la población mestiza, resultado que coincide con el estudio de la Organización Panamericana de la Salud (OPS), titulado "Educación, ingreso y etnia son los factores sociales que más influyen en la salud de los niños, niñas adolescentes y madres" 15, donde se estudia el enfoque diferencial y la desigualdad en la atención en salud.

En cuanto a los antecedentes obstétricos, el 90% de las participantes eran primigestantes (Tabla 1), y al relacio narlo con el estudio de Osvaldo García -"Percepción del parto humanizado en pacientes en el periodo de puerperio"-, el cual fue practicado a 190 pacientes, se evidencia que el 30% eran multigestantes, con edades de 25 a 35 años, y la vía de resolución del embarazo más común fue la cesárea con un 48,9% 16.

Es importante mencionar la definición de violencia obstétrica hecha por Gabriela Almonte García, de acuerdo con la cual "es toda conducta, acción u omisión realizada por el personal de salud que de manera directa o indirecta afecte el cuerpo y los procesos reproductivos de la mujer, lo cual es expresado como un trato deshumanizado o un abuso de la medicación de los procesos naturales". Cuando hace referencia al trato deshumanizado, la autora se refiere a la discriminación, la humillación, o cuando no se brinda asesoría sobre el tipo de parto o atención que requiere la gestante. De igual forma, se refiere a la omisión cuando la gestante o la puérpera no cuentan con información sobre la evolución del parto, el estado de su hijo o hija o las atenciones o actuaciones del personal de salud. En cuento al abuso de la medicación en los procesos naturales, se podría incluir el aumento de los partos medicalizados o cesáreas. También es importante mencionar que la Organización Mundial de la Salud (OMS) planteó que las cesáreas no deberían superar el 15% del total de nacimientos, lo cual no coincide con las estadísticas de los países de América Latina, donde la tasa de cesáreas sobrepasa la cifra antes mencionada y continúa en aumento 17. Lo anterior se puede relacionar con el incremento de las cesáreas a nivel mundial, nacional y regional, que en el caso de la entidad donde se realiza el estudio sobrepasa el 75% del total de nacimientos para el periodo de estudio. Por lo tanto, se puede evidenciar que las puérperas presentan un nivel de violencia obstétrica representado por la falta de infor mación, al no permitírseles escoger el médico tratante ni la institución donde iban a ser atendidas, como tampoco se les brindó educación sobre sus derechos, el tipo de parto y la atención que debían recibir. A ello se suman las manifestaciones del maltrato, las humillaciones y la discriminación sufrida de parte del personal de salud que intervino en dicha atención. De igual forma, el no permitírseles contar con un acompañante en este proceso tan importante para sus vidas, ni tampoco adoptar una posición cómoda durante el parto, no contar con libertad de movimientos y no facilitar elementos de confort para minimizar el dolor, también se manifiesta como deshu manización de parto y violencia obstétrica.

Por lo anteriormente planteado y lo manifestado por Lydia Zacher Dixon en su investigación "Obstetrics in a time of violence: Mexican midwives critique routine hospital practices", de 425 mujeres venezolanas usuarias de los servicios obstétricos, el 49,4% expresó recibir trato deshumanizado durante la atención por parte de los profe sionales de salud, en tanto que el 66,8% aseguró haber recibido atención en salud sin tener en cuenta su consen timiento. El trato deshumanizado se presentó en forma de comentarios sarcásticos, descalificadores, chistes, apodos, críticas por llorar o gritar, e impedimento para manifestar temor o incertidumbre 14. De igual manera, Osmar Figueroa Palomino, en el estudio "Violencia obsté trica: percepción de las usuarias sonorenses realizado durante el 2017 en México", manifiesta que de 45 mujeres atendidas en instituciones públicas, la mayoría manifestó que no se le permitió estar acompañada durante el proceso (71,1%), y a más del 50% se le negó caminar o buscar la posición más cómoda durante su labor de parto 18. Esto coincide con lo evidenciado en el presente estudio y en el de Osvaldo García, donde el 63,2% de las participantes del estudio manifestó que la posición para el trabajo de parto que conocía era la acostada, lo cual se puede relacionar con la poca información que se les brindó sobre posiciones que podían adoptar. En cuanto a la percepción del parto humanizado, el 53,2% aseguró que el médico les explicó, algunas veces, los procedimientos que se iban a realizar, y nunca se le permitió un acompañante al 57,9% 16.

Es frecuente que las puérperas adolescentes no tengan conocimiento sobre el parto humanizado y la importancia que tiene el acompañamiento de una persona signifi cativa durante esta etapa crucial e importante en la vida de una madre y su hijo, más cuando se trata de mujeres que inician esta etapa a tan temprana edad. Se evidenció además que la institución de salud donde se aplicó el estudio no ofrecía la atención del parto humanizado, siendo esta situación anhelada por las gestantes, las cuales quieren vivir este importante momento en compañía de su pareja y su familia. Se encontraron deficiencias en el trato recibido por parte del personal de salud que atendió el parto, y las participantes en su gran mayoría no contaban con información sobre la atención del parto humanizado ♦

## References

[1] Semana Mundial del Parto Respetado.

[2] Arango Urrea JD, Molina Berrío DP, Mejía Merino CM, Faneyra Zapata L (2018). La atención a las madres durante el proceso de parto en algunos servicios de salud de la ciudad de Medellín: un acontecimiento en marcado en el neoliberalismo y la mercantilización de la vida. Gerenc Políticas Salud.

[3] Ariza Olarte C (2012). Soluciones de humanización en salud en la práctica diaria. Enferm Universitaria.

[4] Osorio LAM, Casadiego MAG, Neira AEH, Perez AC (2023). Enfoque dife rencial origen y alcances.

[5] Juscamaita ATV, Castro JAL, Porras M de los ÁL (2013). Parto vaginal des pués de una cesárea, aplicando un punta je al momento del ingreso en un hospital. Rev Peru Ginecol Obstet.

[6] Westbrook LK, Messina DA, Romero M (2016). El parto humanizado: pers pectivas de profesionales en las maternidades públicas de Buenos Aires.

[7] Souza LBC de, Ferreira JE de SM, Oliveira LR de, Chaves AFL, Mon te AS (2021). Percepção das puérperas sobre a assistência humanizada de enfermagem no ciclo gravídico-puerperal: revisão de literatura. Rev Enferm Atual In Derme.

[8] Argote L, Bejarano N, Ruiz de Cárdenas C, Muñoz de Rodríguez L, Vásquez M (2004). Transitando la adolescente por el puerperio: Amenazas, peligros y acciones de protección durante la dieta. Aquichan.

[9] Arteaga Morales BI, Salcedo Daissy L, Lozano Ruiz LT, Prada Prada N, Muñoz FG, Rivera Rodríguez GM Identidades, enfoque di ferencial y construcción de paz.

[10] Llanes G, Bejarano D, Márquez LM, Ponce C, Martínez RM (2018). La huma nización de la atención de enfermería en salud laboral. Rev Enferm Trabajo.

[11] Ministerio de Salud de Colombia Resolución 2626 de 2019.

[12] (2013). World Medical Association Declaration of Helsinki: Ethical Principles for Medical Research Involving Human Subjects.

[13] Ministerio de Salud de Colombia Resolución 8430 de 1993.

[14] Zacher Dixon L (2015). Obstetrics in a time of violence: Mexican midwives cri tique routine hospital practices. Med Anthropol Q.

[15] Documento de Orientación Regional sobre Determinantes Sociales de la Salud en la Región de las Américas.

[16] García-Torres O, Félix-Ortega A, Álvarez-Villaseñor AS (2020). Percepción del parto humanizado en pacientes en periodo de puerperio. Rev Mé dica Inst Mex Seguro Soc.

[17] Cardozo Silva SL, Bernal Roldán MDC (2009). Adolescentes en puerperio y sus prácticas de cuidado. Av Enferm.

[18] Figueroa-Palomino OE, Hurtado-Lagarda R, Valenzuela-Coronado DG, Bernal-Cruz JD, Duarte-Gutierrez CD, Cázares-González FA (2017). Violencia obstétrica: percepción de las usuarias sonorenses. SANUS.

